# Catching Mind Wandering With Pupillometry: Conceptual and Methodological Challenges

**DOI:** 10.1002/wcs.1695

**Published:** 2024-10-22

**Authors:** Claudia Pelagatti, Elvio Blini, Manila Vannucci

**Affiliations:** ^1^ Department of NEUROFARBA, Section of Psychology University of Florence Florence Italy

**Keywords:** mind‐wandering, pupil dynamics, pupillometry, task‐unrelated thoughts, tonic pupil size

## Abstract

Mind‐wandering (MW) refers to the shift of attention away from an ongoing task and/or external environment towards mental contents (e.g., memories, prospective thoughts) unrelated to the task. Physiological measures (e.g., pupil size, EEG, and fMRI) have often been acquired as objective markers for MW states, which has greatly helped their study as well as triangulation with other measures. Pupillometry in particular has been used as a covert biomarker of MW because it is reliably modulated by several distinct processes spanning arousal, emotion, and attention, and it signals attentional lapses. Yet, coupling MW and the measurement of pupil size has led to seemingly contrasting results. We argue that, common to the studies reviewed here, one reason is resolving to the measurement of tonic pupil size, which reflects low‐frequency, slow changes in one's physiological state, and thus implicitly assumes that MW is a static, long‐lasting process. We then additionally focus on three major axes of variability in the reviewed studies: (i) the definition and measurement of MW; (ii) the impact of contextual aspects, such as task demands and individual arousal levels; (iii) the identification and tracking of MW in combination with pupillary measures. We provide an overview of these differences and put forward recommendations for using physiological measures—including, but not limited to, pupil size—in MW research effectively. In conclusion, pupillometry can be a very informative tool for MW research, provided that it is used with the due methodological caution.

## Graphical abstract

We review the studies that use pupillometry as a biomarker for mind‐wandering (MW), highlight the key challenges encountered, and provide recommendations for future research.

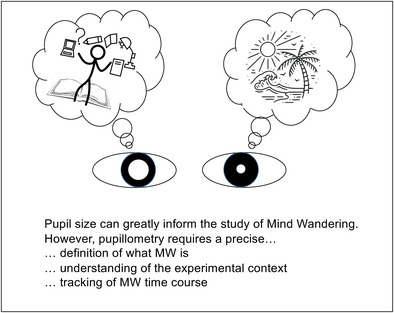

## 1 Introduction

We are attending a crowded seminar, focusing on an evergreen, interesting aspect of human cognition; suddenly, someone's phone rings far away, and the melody brings us memories of someone we used to know with that same ringtone; we start to contemplate reconnecting with this person, though it has been too long; right when we are considering the most appropriate social network to break the ice, the speaker claims “no one knows what attention is”—and we are hooked again. In a situation like this, our attention at some point drifts away from the task at play (attending the seminar) to internal thoughts, which are completely unrelated to the ongoing situation. This “shift in the focus of attention away from the here and now towards one's private thoughts and feelings” (Smallwood and Schooler 2015) constitutes the backbone of mind‐wandering (hereafter MW) episodes, which come however in many forms. For example, the mental contents generated during this mental activity of wandering can be very different, and might include relatively complex personal thoughts, like projections into the past (i.e., autobiographical memories) and the future (e.g., future planning, upcoming events). As we can see from the example above, MW is intrinsically a multifaceted, dynamic process, since it refers to a continuous movement of our attention, from the primary task and the external environment toward internal thoughts. At some point MW starts, it unfolds over time, and then it stops, at least until the next event.

Over the past decade, the investigation of MW has greatly benefited from the adoption of the “strategy of triangulation”, whereby self‐reports, behavioral, and physiological measures are combined, and then used to make inferences about covert mental experiences (Smallwood and Schooler 2015). Among several neurocognitive measures, pupil diameter has become increasingly popular in the research field of MW. Here, we will focus on and review research using pupillometry, highlighting its potential as well as the conceptual and methodological challenges associated with its use in the investigation of MW. We start with briefly introducing and summarizing the rationale behind using pupil diameter as a biomarker of MW (Section 2). For this review, we are going to skip over a complete overview of the physiological and cognitive underpinnings of pupil dynamics, which has been provided by several authoritative reviews (Banks et al. 2015; Binda and Murray 2015a; Einhäuser 2017; Koevoet et al. 2024; Laeng, Sirois, and Gredebäck 2012; Mathôt 2018; Mathôt and Van der Stigchel 2015; Sirois and Brisson 2014; Strauch et al. 2022b; Vilotijević and Mathôt 2023). Rather, we focus on the aspects of pupil dynamics that are more directly relevant to the scientific study of MW. The use of pupillometry in the field of MW has been stimulated by evidence linking attentional lapses (i.e., slow response times, errors in performance) to pupillary measures (Gilzenrat et al. 2010; van den Brink, Murphy, and Nieuwenhuis 2016). Since then, the number of studies on the pupillary correlates of MW has dramatically increased (e.g., Wamsley and Collins 2024; Konishi et al. 2017). However, as we review in Section 3, the patterns of findings are quite mixed: some studies found an increased pupil diameter during MW compared to on‐task states (i.e., participants are focused on the task), whereas others reported the opposite or null findings (reviewed below). This contradictory pattern of results may thus raise doubts concerning the reliability of pupillary measures for investigating MW. However, as we will outline below, there are important methodological and conceptual differences among the studies, and this may largely subtend the different pupillary correlates. More importantly, however, all the studies that we review here are alike in their assessment of one particular pupil size measurement: tonic pupil size. We think that this is a major cause of the reported inconsistencies and thus we selectively review the works that attempt to predict MW states from this specific measure. As we will stress, tonic pupil size reflects slowly changing physiological processes; resolving to this parameter, thus, implicitly assumes that MW is, likewise, long‐lasting and stable, whereas this may not be the case. Finally, we conclude in Section 4 by offering methodological recommendations for using pupillometry in MW studies effectively and outlining the relevance of a conceptual framework based on a dynamic, temporal‐based perspective on MW.

## 2 Pupil Size Reflects Mental States and Dynamic Processes: Potential as Biomarker of MW

The human pupils evolved primarily as a key tool for vision. Their core task is to manage the amount of light reaching the retina at any given moment, and thus optimize visual acuity (Campbell and Gregory 1960; Loewenfeld 1999; Mathôt 2018). However, the pupils also continuously adjust their size following the deployment of distinct endogenous (i.e., mental) processes, in the absence of changes in visual stimulation.

**FIGURE 1:**
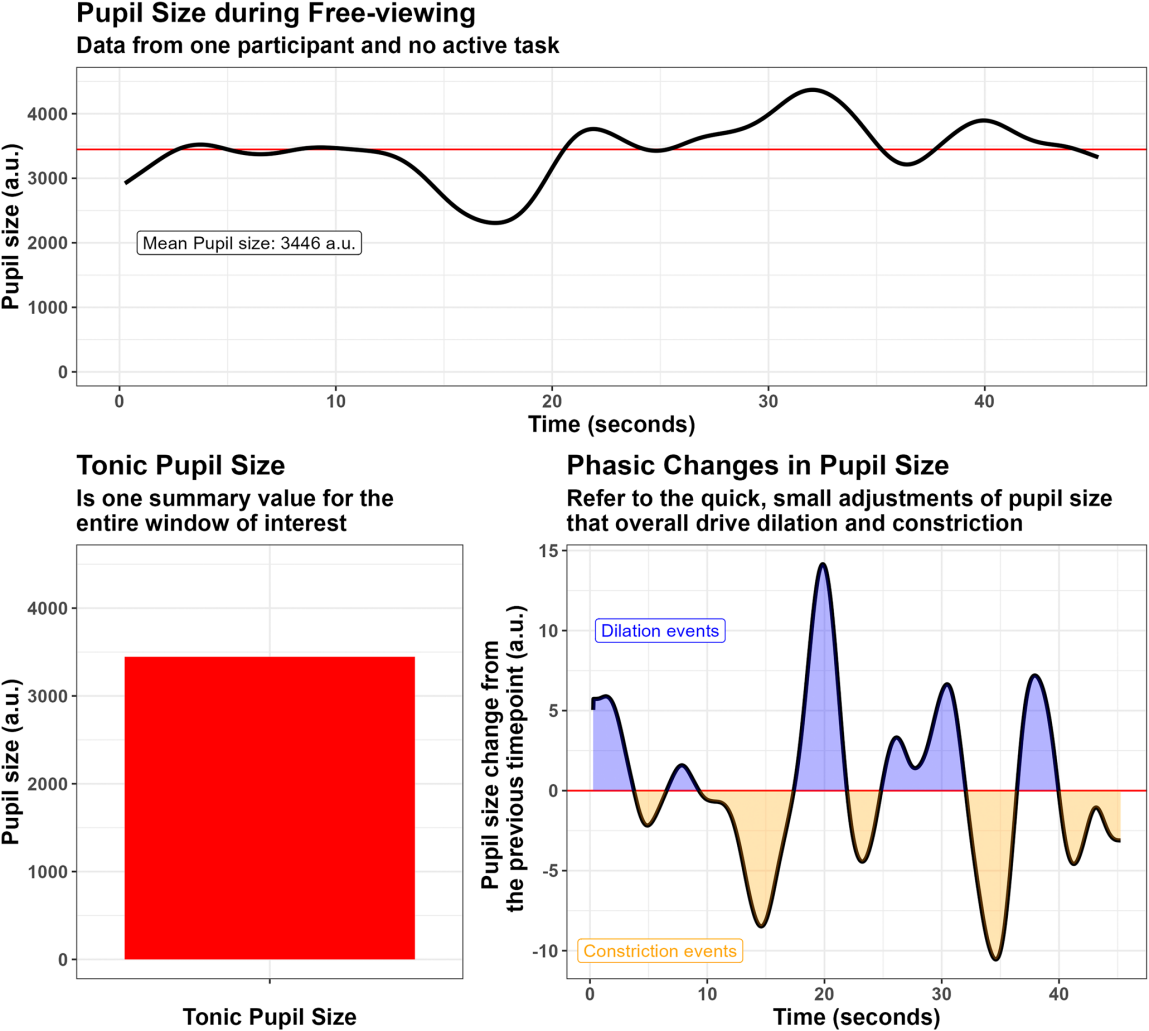
This image is meant to illustrate the difference between tonic and phasic pupil size. The top panel depicts the time course of pupil size in a participant who was passively staring at the screen, without any particular request (i.e., free‐viewing and, perhaps, mind‐wandering). The tonic pupil size in this case is one summary value, for example, the mean, of pupil size in the entire window (or another arbitrary time window); it reflects the relative balance between the sympathetic and parasympathetic autonomic nervous system in this specific moment in time and light conditions, thus in a rather crude way (bottom‐left panel). On the other hand, phasic changes in pupil size return a much more nuanced picture. Here (bottom‐right panel), constriction and dilation events are highlighted, in a bottom‐up fashion, by using the method of Joshi et al. (2016), that is, by assessing when differences between consecutive timepoints crossed zero. Phasic changes in pupil size can be spontaneous, that is, naturally occurring at variable frequencies, or event‐related, when triggered by specific events and cognitive processes (e.g., cognitive load, light reflex). Here we argue that phasic pupil size is a much more appropriate variable to consider when researching mind wandering (MW) and its multifaceted nature because more directly tied to the deployment of these qualitatively different cognitive processes and characterized by higher temporal resolution. Ideally, phasic changes in pupil size should be reconstructed starting from an event identified as triggering MW. This would be the ideal scenario because it shields from arbitrary assumptions about the length and duration of each MW event. That said, assessing spontaneous phasic changes, even if using a fixed‐length time window, is likely to provide more informative and multifaceted summary values than the simple tonic pupil measurement, in that more reflective of the heterogeneous chains of thoughts under consideration.

The size of the pupils under constant light levels reflects directly the balance between the parasympathetic and sympathetic branches of the autonomic nervous system. The parasympathetic branch is involved in homeostatic processes that, at rest, promote relaxation, which is associated with small pupils. The sympathetic branch, on the other hand, regulates bodily functions in conditions of stress, promotes “fight‐or‐flight” responses and behavioral activation, and is associated with large pupils. The first major axis of variation of pupillary signals is therefore to be found in their baseline diameter. The baseline pupil diameter reflects a “tonic” functioning mode, in the sense that it is the result of relatively more stable and long‐lasting physiological states. On the contrary, the fast and transient adjustments that occur at a much quicker pace to finely tune behavior (Aston‐Jones and Cohen 2005) reflect the so‐called “phasic” mode (Figure 1).

Phasic changes in pupil size are usually tiny with respect to the overall variability in pupil size. For context, the human pupils can range between 2 and 8 mm, though most transient effects, however reliable, barely reach 0.1 mm. These changes are largely spontaneous and occur at variable frequencies (e.g., from 0.04 to 2 Hz, Turnbull et al. 2017). In addition, phasic changes that are event‐related have also been described. Similarly to event‐related potentials (ERPs) in electroencephalography, these phasic changes (the signal), if systematic, can only be appreciated once most of the spontaneous baseline pupil fluctuations (the “noise”) are properly controlled for. This typically requires averaging across multiple trials that have been (1) realigned with respect to a putative trigger onset (e.g., the presentation of a stimulus) and (2) realigned with respect to a “starting” pupil size, for example, by subtracting the average pupil diameter during a given time window, so that pupillometry can capture the relative changes with respect to this (supposedly neutral) reference. Both tonic and phasic measurements can inform greatly about the underlying mental states and processes. However, there are nuances in their interpretation that are important to point out, because conflating them may lead to confusion in the results and inconsistent terminology. The most fundamental difference between the two resides in their temporal scale: tonic pupil size measurements likely capture low‐frequency, slow variations, and thus reflect more stable and long‐lasting processes; phasic changes involve high‐frequency, fast changes instead (i.e., happening within few seconds). As a result, phasic changes are more appropriate to evaluate the smaller, quick adjustments that result from the deployment of distinct cognitive processes (Figure 1).

### 2.1 Tonic Pupil Size Reflects one's Overall Physiological State

The tonic functioning mode may be exploited as a good proxy measure of overall alertness and vigilance. Sleep deprivation, for example, is associated with small pupils (Daguet, Bouhassira, and Gronfier 2019; Wilhelm et al. 1998). General anesthesia and sedation, likewise, cause a sharp reduction in baseline pupil size as well as in the variability of pupillary responses (Behrends et al. 2019). Beyond these extreme conditions, even practicing a long, fatiguing task can ultimately lead to a reduction of pupil size (Hopstaken et al. 2015), possibly via depleted sustained attention (Benitez and Robison 2022). Conversely, large baseline pupil size has been associated with an atypically high alertness state, for example in attention‐deficit/hyperactivity disorder (ADHD, Shirama et al. 2020). In a typical scenario, the tonic pupil size is acquired once, under strictly controlled light conditions, and then used for correlations with other variables representing individual traits or temporary states. For example, some research has attempted to link baseline pupil size to fluid intelligence (Tsukahara, Harrison, and Engle 2016), though with much controversy (Robison and Campbell 2023; Unsworth, Miller, and Robison 2021a, 2021b). The main caveat in the usage and interpretation of this measure is that variability is quite large within and between people. The tonic pupil diameter is indeed affected by a very wide range of chemicals, physiological states, and numerous other variables, so it can only be interpreted safely in the context of comparable, strictly controlled settings. For example, circadian rhythms also have an impact on pupil size that does not correlate perfectly with subjective sleepiness, meaning that contextual factors such as the specific hour of the day should be accounted for when using this variable (Daguet, Bouhassira, and Gronfier 2019). To further complicate the matter, personal factors (e.g., individual chronotypes) can also concur in explaining a substantial part of this variability. To summarize, this variable has the potential to inform about MW by providing a coarse assessment of one's physiological state (in terms of vigilance); however, this prognostic value is mostly constrained to states that we believe be relatively stable for a rather long period of time, for example, several seconds or more. If we acknowledge, instead, that MW may encompass ever‐changing, rapidly mutating (even flickering) thoughts, on the other hand, then we should turn to pupillary measures that are better capable of highlighting mental *dynamics*. This is more traditionally accomplished by phasic, event‐related changes in pupil size.

### 2.2 Phasic Changes in Pupil Size Capture Small, Transient Thoughts Dynamics

Phasic changes in pupil size, that is, dilation and constriction events, are largely spontaneous. Here we focus, however, on event‐related responses because more traditionally linked precisely with distinct cognitive processes. Very coarsely, these phasic responses can be grouped into light responses (constriction of the pupils) and psychosensory/cognitive responses (dilation of the pupils).

The first category includes reflexive responses to light. The pupils, starting from 300 ms and up to 1.5 s, constrict to increasing light and then “escape” toward their original state (Mathôt 2018). However, it would be a mistake to believe these responses are completely impervious to cognitive (e.g., attentional) modulations (Binda and Gamlin 2017). For example, the light reflex is increased whenever stimuli are attended, even covertly (Binda, Pereverzeva, and Murray 2013a; Binda and Murray 2015b; Blini and Zorzi 2023; Mathôt et al. 2013), and to the point that this modulation could potentially unveil physiological (Strauch et al. 2022a) and pathological (ten Brink et al. 2023) biases of spatial attention. More generally, “luminance tagging” of stimuli stored in working memory allows one to decode, from pupil constriction, its content, in that prioritizing brighter stimuli increases the light response (Hustá et al. 2019; Koevoet et al. 2024). Likewise, studies have shown that high‐level information about the stimuli—that is, beyond their physical properties in terms of luminance—may act as a cue for an enhanced light reflex. For example, pictures of the sun, despite similar or even lower levels of luminance than control images, trigger increased pupillary constriction (Binda, Pereverzeva, and Murray 2013b; Castellotti et al. 2020). All in all, thus, this line of evidence suggests that pupillometry may indeed be capable to assess and possibly to quantify both the locus and the content of attention. This is directly relevant to the study of mind‐wandering, because MW unfolding has been typically associated with perceptual decoupling from the external environment (Schooler et al. 2011; Smallwood et al. 2011). In other words, the internal mental events that define MW would interfere and detract resources from the processing of external, physical ones, which would then elicit a weaker light reflex as a consequence. There is, however, one additional caveat: when our mind is set free to wander, the pupil dynamics may indeed be decoupled from the external world; however, wandering towards a sunny day at the beach or towards a moonless night in the countryside may yield different pupillary signatures (i.e., relative constriction vs. dilation). In practical terms, the effects of mental contents on pupil size may be tiny to negligible in the overall economy of cognitive processes, which includes far more demanding ones (i.e., working memory load, reviewed below). However, this point further stresses the theoretical appeal of pupillometry in this field of research, on the one hand, and suggests one important caution for future studies: the importance of a rich(er) investigation of mental processes, one that is not limited to binary classifications (e.g., on‐ vs. off‐task), precisely because many of them may yield distinctive effects on pupil size.

The second category of responses may be grouped together because their typical effect on pupil size is that of a sustained dilation. As mentioned above, pupil dilation is one of the signatures of sympathetic activity, and it suggests that the current physiological state is leaning towards increased alertness or focus. Because this part of the spectrum has been characterized as the “fight or flight” mode, it would perhaps not be surprising to note that emotional components play a major role in driving dilation. Indeed, simply viewing or listening to arousing stimuli elicits pupil dilation soon after the initial reflex to light has faded (Bradley et al. 2008; de Winter et al. 2021; Hess and Polt 1960), thus with a rather slow latency but still not as slow as changes captured by tonic pupil size (i.e., within few seconds). For example, viewing images (Bradley et al. 2008) or human faces with changing facial expressions and at different viewing distances (Bogdanova et al. 2022; Dureux et al. 2021) both have been consistently associated with pupil dilation; however, rather than hedonic valence (pleasant vs. unpleasant), the major dimension to which the pupil seems to respond really appears to be emotional arousal—that is, the extent by which the stimulus elicits a heightened physiological activity, regardless of its nature. This is, again, one subtle aspect to keep in mind when adopting pupillometry in the context of MW: results may vary with distracting thoughts that are subjectively felt as being more or less pressing.

Finally, while arousal can be readily associated with the degree by which our (muscular) responses can be fast and vigorous, as would be desirable in a fight or flight situation, it can also be understood as a defining property of several cognitive processes and mental contents. One, influential definition of alertness is precisely that of an overall state of the brain and mind that enables or facilitates an optimal processing of information (Petersen and Posner 2012; Posner 2008). Alertness thus supports and covaries with the deployment of mental effort across tasks of varying difficulty, that is, cognitive load. Indeed, pupillometry gained significant traction as a tool to investigate higher‐level mental processes thanks to landmark studies highlighting increased pupil dilation with increased problem difficulty (Ahern and Beatty 1979; Beatty and Kahneman 1966; Blini, Anobile, and Arrighi 2024; Hess and Polt 1964; Kahneman and Beatty 1966). For example, Hess and Polt (1964) reported that the pupils dilate more strongly for comparatively more difficult arithmetic problems (e.g., 7 × 8 vs. 16 × 23) which, to be solved, require more complex computations than mere fact retrieval. Later on, Kahneman and Beatty (1966) accounted for this effect in terms of different tasks tapping differently on working memory load, by showing robust dilation as a function of the number of digits retained in memory (also see Klingner, Tversky, and Hanrahan 2011). More recent literature has better identified the wealth of high order factors that drive pupil dilation (e.g., divided attention; Lisi, Bonato, and Zorzi 2015; reviewed in Strauch et al. 2022b), thus going beyond the umbrella term of “cognitive load”. All these changes in pupil size have been described for the “phasic” temporal scale, because their onset is generally linked precisely with the onset of the elements to store in memory and their consolidation (Klingner, Tversky, and Hanrahan 2011). However, the same effects are also likely to protract for longer than a few seconds whenever maintenance in working memory is required, for example, for longer chains of thoughts. What is noteworthy in this context is, once again, that while pupil size can inform about the complexity of the unfolding mental operations, its use must be informed by a careful phenomenological assessment of these states, including their putative duration. In other words, wandering towards a chill evening on the sofa, or towards a busy trip home requiring plenty of connecting trains, may both yield distinctive, identifiable pupil dynamics; however, because these dynamics are unlikely to result in equal dilation, a missed characterization of these thoughts may effectively nullify pupillometric measures.

There is one last remark to mention, which refers to the interaction between tonic and phasic pupillary responses: the two pupil modes described above interact in a complex, U‐shaped way (Aston‐Jones and Cohen 2005; Koevoet et al. 2024; Murphy et al. 2011; Murphy, Vandekerckhove, and Nieuwenhuis 2014). This is particularly, but not solely relevant whenever distraction is assessed based on behavioral performance, for example, particularly slow or fast response times within a task. Both very small and very large baseline pupils may be associated with suboptimal performance, for opposing reasons (Murphy et al. 2011; Murphy, Vandekerckhove, and Nieuwenhuis 2014): drowsiness and boredom in the first case, increased distractibility in the latter. When spontaneous, phasic oscillations are the measure of interest, classic models predict that a better performance would be achieved following spontaneous pupil dilation in the case of small baseline pupils, but following pupil constriction in the case of large baseline pupil size (Koevoet et al. 2024); the assumption behind this reasoning is that of an optimal arousal level, located far from the extremes when assessed through pupil size. It is therefore important to stress that, whenever possible, both tonic and phasic pupillary modes should be assessed, because the two likely provide complementary information, not just an overlapping one.

## 3 The Pupillary Correlates of MW Rely on Our Assumptions About MW

In the previous paragraph, we elaborate on why both tonic and phasic measures of pupil size are in principle excellent biomarkers, perfectly capable of enriching our understanding of MW states. We have also stressed, however, that tonic and phasic measures differ along a very important dimension: their temporal granularity. In other words, tonic pupil size reflects slowly‐changing and relatively more stable physiological states; phasic changes in pupil size, on the other hand, are considerably smaller variations that occur very quickly (within few seconds) as an adaptive response to the environment or one's stream of thoughts. Which measure to use, therefore, is directly related to the way we conceptualize MW. If we believe MW to be a long‐lasting, stable, and monolithic process, then tonic measurements are appropriate. If, however, we think of MW as an ensemble of thoughts, occurring in rather fast and unpredictable chains, that is a more dynamic view of MW, then our choice should inevitably include phasic changes in pupil size.

Unfortunately, the two measures are not equally easy to acquire. While it is always possible to record one's pupil size at a given moment, computing event‐related phasic changes requires a crucial, and yet not obvious, piece of information: the onset of the event triggering MW. This is crucial to perform two fundamental operations at once: (1) synchronizing the traces to a “common” generator, so that the traces can be meaningfully averaged; (2) identifying a neutral time window which could serve as a baseline, starting from which we can compute pupil size *variations*. As a reminder, because phasic variations are tiny, this step is often necessary and unavoidable. This leads to the conundrum of MW research through pupillometry: should we stick to an untenable idea of MW, since its onset is so hard to pinpoint with precision?

In this section, we provide a selected overview of the studies that investigated MW by collecting pupil measures. This line of research started more than 10 years ago and has been growing ever since. However, the results are very heterogeneous, with contradictory findings that do not allow us to draw clear conclusions yet. All the studies that we choose to report, however, are similar in their using tonic pupil size measurements. We think, for the reasons outlined above, that this may be the single most important reason why studies have been so seemingly inconsistent, though certainly not the only one.

In one of the first studies in the field, Franklin et al. (2013) recorded the pupil diameter of participants performing a reading task with embedded thought probes. The comparison between the tonic pupil diameter associated with on‐task states and off‐task states (considered as MW) showed that MW states were associated with a larger pupil diameter compared to on‐task states. A similar result (larger tonic pupil diameter associated with MW states) was also reported by Smallwood et al. (2011) by comparing pupil size collected throughout two tasks eliciting a different amount of MW states. Likewise, consistent results have been reported by several other authors (Groot et al. 2021; Jubera‐García, Gevers, and Van Opstal 2020; Oyarzo, Preiss, and Cosmelli 2022; Wamsley et al. 2023). At the same time, however, other studies have reported the opposite outcome. For example, Mittner et al. (2014) asked participants to perform a stop‐signal task, and then occasionally interrupted them with thought probes. In this study, off‐task states (considered as MW) were associated with a smaller baseline pupil diameter with respect to on‐task states. This is not an isolated finding, having been reported by other authors as well (Gouraud, Delorme, and Berberian 2018a, 2018b; Grandchamp, Braboszcz, and Delorme 2014; Huijser, van Vugt, and Taatgen 2018; Konishi et al. 2017; Unsworth and Robison 2016, 2018, Exp 1 and 4; Wamsley and Collins 2024; Whitehead et al. 2021). Finally, the evidence linking tonic pupil diameter and MW states has been inconclusive in several other studies (Groot et al. 2022; Hood et al. 2022; Makovac et al. 2019; Stawarczyk et al. 2020; Unsworth and Robison 2018, Exp 2 and 3; Uzzaman and Joordens 2011; Unsworth and Robison 2018, Exp 2 and 3), thereby confirming that the picture is, indeed, complex and heterogeneous.

This scenario could be discouraging and cast strong doubts about the possibility that pupillometry may reliably point to or predict MW states. Although findings are, indeed, fragmentary, we argue that these discrepancies do not speak as much about the reliability of pupillometry as a tool, but rather about the presence of three major axes of variability in these studies, which in turn reflect more or less implicitly how MW is conceptualized. These axes are to be added to the common feature of the studies that we have reviewed: resolving to a pupillary measure that reflects slowly changing rather stable physiological processes.

### 3.1 Assessment and Identification of MW

The first relevant conceptual and methodological issue refers to the assessment and operationalization of MW. As we can see from Table 1, most of the studies on pupillary correlates of MW used the probe‐caught method. In other words, participants were occasionally interrupted and probed regarding the contents of their experience immediately before the interruption. However, despite the same strategy to catch MW, experiences were collected and categorized in very different ways, with inconsistencies that might have also affected pupillary correlates.

**TABLE 1:** Studies investigating pupillary correlates (tonic Pupil Diameter, PD) of Mind—Wandering (MW).

#	Study	Task	Response modality for thought sampling	Attentional states collected	Time‐window for the analysis of PD	Specific tonic PD results
*MW associated with Smaller PD*						
1	Gouraud, Delorme, and Berberian (2018a)	Obstacle avoidance task (flight simulation).	Thought probes, asking to choose one among different fixed options.	On‐task, something related to the task (these two combined into an On‐task category), MW (explained as “something unrelated to the task”), ED.	10 s before thought probes.	MW associated with smaller PD than on‐task.
2	Gouraud, Delorme, and Berberian (2018b)	Obstacle avoidance task (flight simulation).	Thought probes, asking to choose one among different fixed options.	On‐task, something related to the task (these two combined into an On‐task category), MW (explained as “something unrelated to the task”), ED.	10 s before thought probes.	MW associated with smaller PD than on‐task.
3	Grandchamp, Braboszcz, and Delorme (2014)	Breathing task.	Self‐interruption (whenever participants lose count of their breath).	Focus, unfocus (explained as “losing count of the breath”).	~9 s before or after self‐interruption.	Unfocusing associated with smaller PD than re‐focusing.
4	Huijser, van Vugt, and Taatgen (2018)	Complex spatial working memory task.	Thought probes, asking to choose one among different fixed options.	On‐task, mental elaboration, TRI, ED, MW, inattentiveness.	2 s before thought probes.	MW associated with smaller PD than on‐task.
5	Konishi et al. (2017, Exp 2)	Shape task (0‐back and 1‐back conditions continuously switching).	Thought probes with a continuous slider scale, (from not at all on‐task, or completely off‐task, to completely on‐task).	On‐task, Off‐task (explained as “something unrelated to the task”).	~3.5 s before thought probes.	Off‐task (past/intrusive) associated with smaller PD than on‐task.
6	Mittner et al. (2014)	Stop‐signal task.	Thought probes with 5‐point Likert scale (ranging from task‐independent to task‐centered).	On‐task, Off‐task (explained as “task‐independent thoughts”).	1 s before task stimulus onset.	Off‐task associated with smaller PD than on‐task.
7	Ozawa et al. (2022)	Finger‐tapping task (constant and non‐constant).	Thought probes with 7‐point Likert scale for Attention (ranging from off‐task to on‐task); and asking to choose one among different fixed options for Conceptual division.	(1) Attention: On‐task, Off‐task (2) Conceptual division: On‐task (explained as “task focus or thinking of nothing at all”), TRI, unpleasant task‐unrelated thoughts, neutral task‐unrelated thoughts, pleasant task‐unrelated thoughts.	Different 5‐s periods along the task (from 5 s before stimulus, to 20 s after stimulus).	Larger PD was positively correlated with on‐task. Note: this is a between‐participants correlation
8	Unsworth and Robison (2016)	Psychomotor vigilance task.	Thought probes, asking to choose one among different fixed options.	On‐task, TRI, ED, BLANK, MW.	2 s before task stimulus.	MW associated with smaller PD than on‐task.
9	Unsworth and Robison (2018)	Psychomotor vigilance task, Stroop task.	Thought probes, asking to choose one among different fixed options.	On‐task, TRI (excluded for analysis), MW, ED, BLANK (last three combined into an Off‐task category for analysis).	2 s before task stimulus.	Off‐task associated with smaller PD than on‐task.
10	Unsworth & Robison (2018, Exp 1)	Psychomotor vigilance task, Stroop task.	Thought probes, asking to choose one among different fixed options.	On‐task, TRI, ED, BLANK, MW.	2 s before task stimulus.	MW associated with smaller PD than on‐task.
11	Unsworth & Robison (2018, Exp 4)	Psychomotor vigilance task with two conditions: fixed 2‐s wait time, and fixed 8‐s wait time.	Thought probes, asking to choose one among different fixed options.	On‐task, TRI, ED, BLANK, intentional, unintentional (last two combined into MW).	2 s before task stimulus.	2 s fixed condition: MW associated with smaller PD than on‐task. 8 s. fixed condition: no significant difference between MW and on‐task.
12	Wamsley and Collins (2024)	SART (numbers stimuli, long SOA) with two conditions (High cognitive load, Low cognitive load), and Task‐free condition.	Thought probes for subjective self‐reports, asking to choose one among different fixed options.	The classifier of states on the basis of EEG, pupil, RTs and subjective self‐reports (external task‐related, external task‐unrelated, internal task‐related, internal task‐unrelated). States individuated: On‐line, Offline 1, Offline 2.	—	SART: Offline 1 associated with smaller PD than Online or Offline 2. Task free: Offline 1 and Offline 2 associated with smaller PD than Online.
13	Whitehead et al. (2021, Exp 2)	Cued task‐switching paradigm.	Thought probes, asking to choose one among different fixed options.	On‐task (explained as “being focused on some aspects of the task at hand”), Off‐task trying (explained as “unintentional task‐unrelated thoughts”), Off‐task not trying (explained as “intentional task‐unrelated thoughts”).	500 ms before task stimulus onset.	“Off‐task—not trying” associated with smaller PD than OT.
*No significant differences between pupil size between MW and on*‐*task states*						
14	Groot et al. (2022)	Finger‐tapping sequence generation task.	Thought probes with 6‐point Likert scale (ranging from clearly on‐task to clearly off‐task).	On‐task (explained as “task‐related thoughts or task focus”), Off‐task (explained as “task‐unrelated thoughts”).	Trial‐wise summary statistics.	No significant difference between off‐task and on‐task.
15	Hood et al. (2022)	Prosaccade and antisaccade task.	Thought probes, asking to choose one among different fixed options.	Task, task‐performance (these combined into an On‐task category), everyday stuff, current state of being, personal worries, daydreams, other (all these others combined into an Off‐task category).	First 30 ms of the fixation screen before task stimulus.	No significant difference between off‐task and on‐task.
16	Huijser et al. (2020)	SART (words stimuli, with embedded current concerns, long ISI).	Thought probes, asking to choose one among different fixed options.	Attentional state: On‐task, TRI, ED, BLANK, concern‐related thought (personal matters), MW (task‐unrelated matters; last two combined together into a MW category)	500 ms before task stimulus onset.	No significant difference between MW and on‐task.
17	Jubera‐García, Gevers, and Van Opstal (2020, Exp 2)	SART (numbers stimuli, long ISI).	Thought probe with dichotomous response for content (on‐task or off‐task).	Content: On‐task, Off‐task (explained as “task‐unrelated thoughts”)	500 ms before task stimulus onset for 8 trials before thought probes (distance from thought probes = 21.6 s).	No significant difference between off‐task and on‐task.
18	Makovac et al. (2019)	Low‐demand visuo‐motor tracking task.	Thought probes, with three horizontal scales, ranging from 0 to 100 (How much engaged in: OT, PC, MW).	On‐task, perseverative cognition, MW (explained as “thinking about different things or events, without being stuck in one particular thought”).	3‐s before target appearance.	Perseverative cognition associated with larger PD, but not self‐reported MW.
19	Robison and Unsworth, (2019, Exp2)	Visual working memory task.	Thought probes, asking to choose one among different fixed options.	On‐task, TRI (excluded for analysis), intentional MW, spontaneous MW, ED, BLANK (last four combined into an Off‐task category for analysis).	1‐s fixation screen before task stimulus.	No significant difference between off‐task and on‐task.
20	Stawarczyk et al. (2020)	SART (numbers stimuli, long ISI).	Thought probes, asking to choose one among different fixed options.	On‐task, TRI, ED, BLANK, MW.	A whole block (from 30 to 90 s) of the task, before thought probes.	MW associated with smaller PD than on‐task, but this difference was no longer significant when sleepiness was added in the model.
21	Unsworth & Robison (2018, Exp 2)	Psychomotor vigilance task + instruction to give a speech in front of a video camera after the task.	Thought probes, asking to choose one among different fixed options.	On‐task, TRI, ED, BLANK, MW.	2 s before task stimulus.	No significant difference between MW and on‐task.
22	Unsworth & Robison (2018, Exp 3)	Psychomotor vigilance task with two conditions: variable wait time, and 5‐s. fixed wait time.	Thought probes, asking to choose one among different fixed options.	On‐task, TRI, ED, BLANK, past, future, current state (last three combined into MW).	2 s before task stimulus.	No significant difference between MW and on‐task. No interaction Condition*Attentional state (but in the variable condition PD was significantly smaller in MW than in on‐task).
23	Uzzaman and Joordens (2011)	Reading task.	Thought probes with dichotomous response (zoning out yes or not).	On‐task, Off‐task (explained as “zoning out”).	5 s. before thought probes.	No significant difference between off‐task and on‐task.
*MW associated with Larger PD*						
24	Franklin et al. (2013)	Reading task.	Thought probes with dichotomous response (MW yes or not).	On‐task, Off‐task (explained as “wandering mind”).	10 s before thought probes.	Off‐task associated with larger PD than on‐task.
25	Groot et al. (2021)	SART (numbers stimuli, short ISI).	Thought probes with 4‐point slidebar (ranging from off‐task to on‐task).	On‐task, Off‐task (explained as “attention not primarily focused on the task or environmental distractions but on internal processes such as memories or personally relevant thoughts”).	Trial‐wise summary statistics.	Off‐task associated with larger PD than on‐task.
26	Jubera‐García, Gevers, and Van Opstal (2020, Exp 1)	SART (numbers stimuli, long ISI).	Thought probe with dichotomous response for content (on‐task or off‐task) and 4‐point scale for intensity of focus.	(1) Content: On‐task, Off‐task (explained as “task‐unrelated thoughts”) (2) Intensity of focus.	500 ms before task stimulus onset for eight trials before thought probes (distance from thought probes = 21.6 s).	Off‐task associated with larger PD than on‐task.
27	Oyarzo, Preiss, and Cosmelli (2022)	Reading task.	Thought probes with dichotomous response (on‐task or distracted) + self‐interruption (when distracted).	On‐task, Off‐task (explained as “distracted by something else”).	5 s before thought probes or before self‐interruptions.	Off‐task associated with larger PD than on‐task.
28	Smallwood et al. (2011, Exp 2–3)	Working memory task (to collect On‐task states), Choice reaction time task (to collect Off‐task states).	No thought sampling, comparing two task (WM task, CRT), assuming CRT as associated with more offline thoughts.	On‐task, Offline thoughts (explained as “task‐unrelated events and abstract task‐unrelated thoughts”).	1.5 s before task stimulus.	Offline thoughts associated with larger PD than on‐task.
29	Unsworth, Robison, and Miller (2019)	Baseline eye‐fixation.	Post‐task questionnaire, asking to circle the categories that best describe what participants were previously thinking at.	On‐task, ED, BLANK, drowsy, past negative, past positive, future negative, future positive (last four combined into a MW category).	30‐s periods of the 5‐min eye‐fixation task.	Positive correlation (*r* = 0.15) between PD and MW. Note: this is a between‐participants correlation
30	Wamsley et al. (2023)	SART (numbers stimuli, long SOA).	Thought probes for subjective self‐reports, asking to choose one among different fixed options.	Classifier of states based on EEG, pupil, RTs and subjective self‐reports (external task‐related, external task‐unrelated, internal task‐related, internal task‐unrelated). States individuated: On‐line, Offline 1, Offline 2.	1‐s window ending 200 ms before stimulus onset.	Offline 2 associated with larger PD than Offline 1 or online. Offline 1 associated with marginally larger PD than online.

*Note*: In the table, the studies are grouped according to the observed pupillary pattern, and then alphabetically. Please note that few studies assessed both tonic and phasic pupil size, but here we specifically focused on the first.

Abbreviations: ED = external distraction; MW = mind wandering; PD = pupil diameter; SART = sustained attention to response task; TRI = task‐related interference.

In some studies, researchers just focused on the comparison between “being on‐task” (i.e., completely focused on the ongoing task) and “being off‐task” (i.e., unfocused on the task, focused on something else) (e.g., Groot et al. 2021; Mittner et al. 2014; Smallwood et al. 2011). The modalities used for collecting these responses range from a dichotomous response (e.g., “MW yes vs. MW no” in Franklin et al. 2013; “zoning out yes vs. zoning out no” in Uzzaman and Joordens 2011; “on‐task vs. distracted by something else” in Oyarzo, Preiss, and Cosmelli 2022), a Likert scale with a variable number of points (e.g., 5‐point Likert scale ranging from “task‐independent” to “task‐centered”, in Mittner et al. 2014–4‐point Likert scale ranging from “on‐task” to “off‐task”, in Groot et al. 2021; 6‐point Likert scale ranging from “clearly on‐task” to “clearly off‐task”, in Groot et al. 2022; 7‐point Likert scale ranging from “off‐task” to “on‐task”, in Ozawa et al. 2022) or a continuous slider scale ranging from “completely off‐task” to “completely on‐task” (Konishi et al. 2017).

We believe that this broad distinction between “on‐task” and “off‐task” (focused on something else, unrelated to the task) might be quite problematic in the investigation of MW and its pupillary correlates. Specifically, the category of “off‐task” (or “offline” in Smallwood et al. 2011), while certainly characterized by the lack of focus on the task at hand, may indeed comprise different attentional states, namely different types of distraction, and not exclusively MW. For example, someone might report being “off‐task” when distracted by stimuli in the environment (e.g., “I could hear the steps outside the door”), by thoughts related to bodily sensations (e.g., “my nose started itching”), by a personal future plan (e.g., “I was thinking about my upcoming exam”), or even by a temporary absence (“I wasn't thinking at something in particular”). Although all these states can be considered “off‐task” and all these contents can be defined as task‐unrelated, they indeed refer to different attentional states. In keeping with this reasoning, few researchers have introduced and applied a more fine‐grained categorization of attentional states. In a pioneering study, Stawarczyk et al. (2011) introduced a classification of ongoing conscious experiences that distinguishes “on‐task” from “task‐related interference” (i.e., thinking about task‐related matters, such as one's own performance or the task's duration); external distraction—distraction by physical sensations (i.e., being distracted by sights/sounds or physical sensations such as hunger or thirst) and MW (i.e., thinking about things unrelated to the ongoing task). This categorization has been slightly modified by Unsworth and Robison (2016) (see also Pelagatti, Binda, and Vannucci 2018, 2020) with the inclusion of the additional state of blank mind (i.e., states without a specific mental content, but also including drowsiness and lack of alertness according to some authors). When this categorization was applied, evidence showed that the different attentional states indeed behave differently. For example, Unsworth and Robison (2016) showed that different types of lapses of attention can be associated with a different baseline pupil size: smaller for inattentive and MW states, but larger for external distractions when compared with on‐task reports.

Altogether these findings demonstrate that under the umbrella term of “off‐task” there are dissociable cognitive experiences and emphasize the importance of assessing them separately.

Although, over the years, an increasing number of studies have been using this categorization, there are still some inconsistencies in its application. For example, few studies have included some sort of thoughts, such as task‐related interferences, into the “on‐task” category (e.g., Gouraud, Delorme, and Berberian 2018a, 2018b; Groot et al. 2022; Whitehead et al. 2021). Task‐related interferences are thoughts about task stimuli, task performance, or task duration, and they indeed differ from the pure sense of focusing. Combining task focus and task‐related interferences together may thus contribute to confound the comparison between on‐task and off‐task states.

Most of the studies that employed the categorization developed by Unsworth and Robison (2016) used a “fixed multiple choice” as a modality of response at probes, thereby asking participants to select one among different fixed options to describe their experience prior to the probe, as on‐task, MW, external distractions, task‐related interferences, blank mind (in Stawarczyk et al. 2020; Unsworth and Robison 2016).

Recently, a few authors introduced an open question modality at probes, asking participants to provide a short description of their thoughts, if any (Pelagatti, Binda, and Vannucci 2018, 2020; Plimpton, Patel, and Kvavilashvili 2015). All thoughts recorded by participants are therefore independently coded by trained judges who categorize the reported mental contents following the schema of categorization reported above.

Although both procedures have pros and cons, having short descriptions of the thoughts might be helpful to get more information about the contents of MW episodes, and their level of complexity.

Since the pupils are sensitive to the cognitive load associated with the ongoing mental activity, we believe that knowing about the contents generated during MW might better frame the information provided by the pupillary response. Likewise, because pupil size does change as a function of a number of variables, including emotional arousal, implied brightness, and so on (as reviewed above), a more nuanced description of mental states could ultimately be warranted to understand which processes are reflected in the observed pupillary correlates.

### 3.2 The Role of Task Context in MW

The second broad issue concerns the context in which MW arises and it is assessed, starting from the ongoing task. Although episodes of MW may occur potentially during any kind of task, several studies have shown that certain characteristics of the ongoing task may affect both the frequency and the properties of MW. It is well‐known that the difficulty and cognitive load implied by a task drive the frequency of MW (e.g., Rummel and Boywitt 2014; Smallwood, Nind, and O'Connor 2009) its temporal focus (e.g., Smallwood, Nind, and O'Connor 2009) and the spontaneity of thoughts (Seli, Risko, and Smilek 2016). Moreover, some aspects of the task may also have an influence on participants' attitudes, such as motivation levels and interest, and these dimensions modulate MW in turn (e.g., Seli et al. 2019; Unsworth and McMillan 2013). Therefore, the choice of the task to administer is not trivial, as it may elicit very different emotional states, MW types, and pupillary correlates.

Nevertheless, as clearly shown in Table 1, there is a high heterogeneity of tasks in studies comparing pupil diameter between MW and on‐task states. For example, few authors probed attentional states while participants performed reading tasks (e.g., Franklin et al. 2013; Oyarzo, Preiss, and Cosmelli 2022; Uzzaman and Joordens 2011). Other authors preferred, instead, psychomotor vigilance tasks, in which participants were presented with a string of visual stimuli (e.g., zeros) and had to press a button as soon as the numbers started to count up (e.g., Unsworth and Robison 2016, 2018). Other studies employed a stop‐signal task (e.g., Mittner et al. 2014) in which participants had to respond to the orientation of rapidly presented arrows (pointing to the left or to the right) and to withhold the response when perceiving an auditory stimulus, or the more automatic SART with digits (e.g., Jubera‐García, Gevers, and Van Opstal 2020) or letters (e.g., Huijser et al. 2020), and at a different task‐pace (long ISI in Stawarczyk et al. 2020; short ISI in Huijser et al. 2020).

In summary, MW and pupil size have been investigated during the performance in tasks that differ both in terms of the amount of (prolonged) cognitive effort and the specific cognitive processes involved (e.g., working memory, sustained attention). In these different scenarios, different baseline, and tonic pupil diameters can be expected. Ultimately, this may underlie many contradictory results obtained when comparing MW versus on‐task reports, as several authors have already stressed (e.g., Konishi et al. 2017; Unsworth and Robison 2018; Wamsley and Collins 2024).

For example, Konishi et al. (2017) demonstrated that the average, tonic pupil diameter collected during the 0‐back condition was smaller relative to the more demanding 1‐back task. This can determine, in turn, the direction towards which the mind (and pupils) may more likely wander when off‐task: pupils would be more likely to wander towards more demanding thoughts in boring tasks, but towards less demanding and “refreshing” thoughts when disengaging from very challenging ones. Think about the most complex mental operations you can imagine, say integrals. Imagine a cynical researcher asking you to perform heavy‐duty math for hours. Now imagine yourself taking a mental break, utterly exhausted: would you more likely blank out for a moment or end up with an even more challenging mental diversion?

In a similar vein, Unsworth and Robison (2018) show, in four experiments, that tonic pupil diameter varied as a function of the experimental manipulation of task context and the associated level of arousal. Specifically, depending on the task context and the extent to which it promotes an external or internal orientation of attention, there might be different MW states associated with different arousal (and alertness) levels, and pupillometry might reveal this heterogeneity.

In the study, the authors found two different patterns of pupillary correlates. The condition of high task demands promoted focused external attention. In this case, the few MW states reported at thought probes were associated with lowered arousal, and thus smaller tonic pupil diameter compared to on‐task reports. However, the less demanding task promoted an *internal* focus of attention. In this case, conversely, the amount of MW increased and was associated with an intermediate level of arousal, similar to the one associated with on‐task. In this condition, tonic pupil diameter was similar for MW and on‐task reports.

Finally, Stawarczyk et al. (2020) also contributed to shed light on the role of individuals' arousal levels, by showing the importance of taking their drowsiness into account when measuring pupil size across attentional states. These authors collected pupil measures while participants performed the SART with embedded thought probes. At each probe, the Karolinska Sleepiness Scale (KSS; a 9‐point Likert scale assessing subjective sleepiness, Akerstedt and Gillberg 1990) was also presented. Results showed that MW was associated with smaller mean pupil diameter than on‐task states, but this difference was no longer significant when KSS scores were considered as covariates. This suggests that the results, in this case, may be driven by drowsier participants, who were possibly more susceptible to MW episodes.

### 3.3 Catching and Tracking MW

The third issue to deal with, after defining MW and developing an appropriate task procedure, is to decide how to track MW and when and how to analyze pupil diameter. As outlined in Section 2, this passage is what really informs about the underlying assumptions about MW: is MW a rather stable and long‐lasting state of the mind, or rather a dynamic chain of loosely‐related and flickering thoughts?

Most studies recorded both MW and pupil diameter throughout the same task. Few exceptions, however, acquired a “cold” measure of pupil size which was correlated with post‐task questionnaires used to collect task‐unrelated thinking (Unsworth, Robison, and Miller 2019). Another common strategy is to compare pupil size between two tasks commonly associated with a different amount of task‐unrelated thinking (which may not, however, be assessed concurrently to pupillary recordings; Smallwood et al. 2011). In the vast majority of the studies recording both MW and pupil diameter throughout the same task, attentional states are collected by using the probe‐caught method, sometimes with thought probes triggered by behavioral indices (Makovac et al. 2019; Robison and Unsworth 2019), or the self‐caught method, either alone (Pelagatti, Binda, and Vannucci 2020), in combination with the probe‐caught (Oyarzo, Preiss, and Cosmelli 2022), or with other procedures (Grandchamp, Braboszcz, and Delorme 2014). The self‐caught method requires participants to press a button when they realize that their attention is disengaged from the task and to self‐report their experience. Grandchamp, Braboszcz, and Delorme (2014) introduced a variation of this method in which participants perform a breathing task and have to press a button whenever they lose their breath count, assuming MW when these interruptions occur. One general problem of this approach with respect to physiological variables, including pupil size, lies in the cognitive operations that are necessary to notice MW (i.e., meta‐awareness) and then provide a motor response. This is likely to be a confound in studies attempting to predict MW from biomarkers if motor and premotor activity covary with states identified as MW (i.e., if control states do not present such signature activity), because premotor activity is well captured by pupil size (see, e.g., Eisenberg and Zacks 2016). This is particularly the case if physiological measures are aligned to and compared in the near proximity of this motor response as opposed to, for example, being aligned to putative MW triggers that occurred several seconds before that.

At any rate, when attentional states and pupil diameter are collected during the same task or trial, one common problem is to choose how to relate pupil size to the specific attentional state. In other words, once a MW state is identified, its putative time course must also be defined. This is not obvious but has the immediate direct consequence of driving the choice of the time interval in which pupil size is calculated. All uncertainties in establishing the onset and duration of the attentional state therefore transfer directly to uncertainty about the most appropriate pupillary recordings to use. As reviewed above, the great majority of studies chose to analyze the tonic pupil diameter in pre‐established time windows. Two remarkable problems arise with this common choice.

The first problem concerns the high heterogeneity in the time windows used in the studies to compare on‐task and MW episodes (see Table 1). For example, Uzzaman and Joordens (2011) used thought probes to collect attentional states while participants performed a reading task: they compared the tonic pupil diameter in the 5‐s time window before thought probes associated with either on‐task or off‐task states. Franklin et al. (2013), despite using a reading task as well, chose twice the amount of time for the windows prior to thought probes (i.e., 10‐s time‐windows). Other authors, instead of analyzing pupil diameter immediately before thought probes, took the baseline pupil diameter before and/or after the presentation of task stimuli that were followed by on‐task or off‐task reports (e.g., Mittner et al. 2014; Unsworth and Robison 2016, 2018; Wamsley and Collins 2024; Whitehead et al. 2021). For example, Unsworth and Robison (2016) computed the average pupil diameter during the fixation screen lasting 2 s before the presentation of the stimulus, which was occurring more than 3.5 s before thought sampling. Finally, Stawarczyk et al. (2020) extracted the mean pupil diameter over the duration of each block in the SART, lasting from 30 to 90 s, and ending with a thought probe. All in all, the choice of a given time window appears very arbitrary, and this heterogeneity makes it difficult to compare the results of different studies. Still, this could be arguably considered a minor problem, as the second one is far more debilitating. The second problem is the very idea of a “fixed” time window, identical for all reports and across participants, and the reliance on the tonic dimension of pupil size. The underlying assumption behind this choice is that the duration of MW episodes is relatively constant, and MW episodes are internally very similar and long lasting. Indeed, this is not the case: MW episodes may vary in duration, spanning from a very few to several seconds (Grandchamp, Braboszcz, and Delorme 2014; Klinger 1978; Pelagatti, Binda, and Vannucci 2020). In a resting state study, Vanhaudenhuyse et al. (2011) reported evidence for a periodic shift from external to internal awareness occurring, on average, every 20 s (0.05 Hz ± 0.03 Hz). Other studies report mean durations of MW episodes between 10 and 20 s, but with standard deviations that are almost as large (e.g., Bastian and Sackur 2013; Voss, Zukosky, and Wang 2018) thus pointing to a stark variability that is not compatible with the choice of using a constant time window. It seems unlikely that the fixed time‐window strategy, however convenient, may reflect the precise timing of every and each MW episode. Rather, pupillary traces may not cover the whole duration of MW (when the fixed time‐window is shorter than the actual duration of the MW state), or, worse, they may cover states that are temporally contiguous to MW but do not overlap (when the fixed time‐window is longer than the actual duration of the MW state). Choosing to rely on the tonic functioning mode, furthermore, only reiterates this assumption by analyzing a measure that reflects low‐frequency changes of stable physiological states. Again, these methodological choices are very likely to have an impact on the reported results, including their being often contradictory.

## 4 Future Perspectives and Recommendations

Based on the points raised in the previous sections, here we suggest some methodological recommendations to use pupillometry in MW research effectively, or at least consistently. Our suggestions are grouped according to the three main issues outlined above.

### 4.1 Recommendation 1: A More Precise Description of the Experience of MW

First of all, we should recognize that MW is only one of many possible task‐unrelated attentional states that participants may experience in a task (e.g., Stawarczyk et al. 2011). Consequently, it is of paramount importance to define each attentional state clearly when collecting participants' reports, so that these states are distinguishable. For example, including only states of focus into the “on‐task” category (e.g., Unsworth and Robison 2016) is not the same as including both states of focus and thoughts about performance/mistakes (e.g., Gouraud, Delorme, and Berberian 2018a). Similarly, defining and assessing MW in terms of being “off‐task” or being “distracted by something” (Oyarzo, Preiss, and Cosmelli 2022) is probably inadequate because of the high heterogeneity of distractions included under these terms. Moreover, as we reviewed above, in the studies with probe‐caught methods, different response modalities have been used. In this regard, studies by Weinstein (2018) [see also Weinstein, de Lima, and van der Zee 2018] have shown that the phrasing of the questions at probes, as well as the response modality, may influence how the experience of MW is reported. The use of open questions at probes (e.g., “What were you thinking about just immediately before the probe?” or “Where was your attention focused at prior to the probe?”) with independent and expert judges trained in the classification of reports, may be particularly helpful to have a deeper understanding of the experience of MW, getting to know the kinds of mental contents generated during this state (e.g., autobiographical memories, prospective memory, future scenarios) and their complexity. This is unquestionably demanding for MW researchers. However, as reviewed above, pupil size is also very sensitive to the specific processes at hand, including their content. For example, the amount of cognitive load and emotional arousal does matter for pupil size. Thus, if the objective is to make sense of this measure, or to probe it as a viable biomarker for MW states, a more nuanced description of subjective experience is warranted. Furthermore, adopting this procedure may help to reduce misclassification of mental states and increase the comparability between studies.

### 4.2 Recommendation 2: Minding Contextual Factors

The second methodological recommendation concerns contextual factors. As discussed above, both the cognitive load implied by the task and participants' arousal level have a critical influence on pupil diameter. We would therefore suggest including the assessment of these variables in future studies. On the one hand, the nature of the task and the perceptual and cognitive processes needed to perform it should be clearly documented. On the other hand, the subjective task difficulty should be ideally measured as well, as the effort that participants put in performing the task. More generally, participants' motivation and level of interest would also need to be probed quantitatively. Measuring arousal and drowsiness levels, not only at the beginning of the session but throughout the course of the whole task, may be also beneficial because lapses of attention may happen at various levels of arousal (Lenartowicz, Simpson, and Cohen 2013), though not necessarily with the same pupillary outcomes. Moreover, given that circadian rhythms also have an impact on pupil size (see Section 2), it would be useful to account for the specific hour of the day and the individual chronotypes when running a study. The few studies that have already included a number of these variables into their design have clearly shown their impact on the experience of MW and its pupillary correlates (Unsworth and Robison 2018; Stawarczyk et al. 2020). Collecting all this information, whenever feasible, in the same setting, is important because the content of MW and the context in which it emerges are key to its relation with pupil size (Konishi et al. 2017; Pelagatti, Binda, and Vannucci 2020).

### 4.3 Recommendation 3: Tracking the Time‐Course of MW

Finally, the third aspect, which we believe is most important to start investigating, is the modality for tracking MW and then analyzing the corresponding pupil diameter. In the previous section, we exposed the heterogeneity of time windows used, as well as the risks tied to the use of fixed‐duration and pupillary indices that are more reflective of stable, slow processes. In this respect, a more dynamic approach to the investigation of MW would be highly warranted.

For a long time, MW has been considered as stimulus‐independent and self‐generated (e.g., Antrobus 1968; Smallwood 2013), making it difficult to investigate the dynamics of thoughts' flow starting from its beginning. However, we have recently understood that a great part of MW may be triggered by external triggers (e.g., Faber and D'Mello 2018; Maillet, Seli, and Schacter 2017; Plimpton, Patel, and Kvavilashvili 2015; Song and Wang 2012; Vannucci, Pelagatti, and Marchetti 2017); these triggers could be leveraged upon and used as anchor points for exploring the initial dynamics of the thoughts flow. Specifically, experimental studies on MW ran in a laboratory setting have shown that both task‐irrelevant (McVay and Kane 2013; Niedźwieńska and Kvavilashvili 2018; Pelagatti, Binda, and Vannucci 2018; Plimpton, Patel, and Kvavilashvili 2015; Vannucci, Pelagatti, and Marchetti 2017) and task‐relevant external stimuli (Faber and D'Mello 2018; Maillet and Schacter 2016; Maillet, Seli, and Schacter 2017) might indeed act as triggers for MW and thus serve as anchoring points for pupillometric measures.

The opportunity to identify the starting point of a MW episode via these triggers enables us to create, for example, adjustable time‐windows, tailored to both MW types and participants (e.g., Pelagatti, Binda, and Vannucci 2018). Thus, thought triggers should be carefully evaluated when presenting participants with thought probes. By doing so, we could greatly enhance the precision by which we can match each MW episode with the associated pupillary recordings. Moreover, improving our identification of the starting point of MW raises the possibility to better capture the entire dynamics of pupil changes, that is those that occur from MW ignition and continue afterward (Smallwood 2013), through the maintenance.

In this regard, we (Pelagatti, Binda, and Vannucci 2018) recently employed a joint behavioral‐pupillary paradigm to track the dynamics of MW triggered by external stimuli, namely task‐irrelevant verbal cues presented during a monotonous vigilance task (for more details on this task, see Plimpton, Patel, and Kvavilashvili 2015). Specifically, by capitalizing on the MW triggering effects of these cues during the task, we compared, trial by trial, the time course (over 6 s) of pupil diameter observed in distinct conditions. The conditions that are relevant here are those defined by a verbal cue reported by the participant as the trigger of a spontaneous MW episode (MW trigger) at probe, or after a verbal cue (with similar emotional content) which was followed by an on‐task report at the probe, for which pupil size can then be assessed directly. Indeed, trials can then be temporally realigned to the onset of the cue later identified as triggering or nontriggering an MW event; the average pupil diameter during the reference event (the presentation of the word) can then serve as an ideal baseline from which computing phasic changes in pupil size. Adopting this strategy led us to conclude that pupil diameter increased more when the cue‐word triggered MW compared to other cues that were followed by on‐task reports; this held true despite the similar emotional content between categories. These findings were later replicated and extended to the self‐caught rather than a probe‐caught procedure to assess the occurrence of spontaneous MW episodes (Pelagatti, Binda, and Vannucci 2020). All in all, this shows that tracking the time course and dynamics of MW can be feasible, although few methodological cautions are required. These approaches have the promising potential to reduce our uncertainty about the onset of MW (its ignition, Smallwood 2013), and then to better describe its unfolding over time with (phasic) pupillary measures that are better apt for this task (as reviewed above).

## 5 Conclusions

Pupillometry has been widely adopted in MW research as a physiological marker, though contradictory results have cast doubts about its usefulness and reliability. We have reviewed why and how pupillary measures can in principle be instead very informative and effective to this aim, once some methodological aspects are duly considered. Specifically, we selectively reviewed studies that used tonic pupil size to assess MW states, driven by the assumption that this can subtend a large share of the reported variability. We therefore claim that this choice does indeed imply an untenable assumption about MW, that is that MW has a relatively long and stable nature, and thus may be particularly susceptible to physiological noise and variability, hence inconsistencies in the results. Furthermore, we have identified important axes of variations in current MW research that require renewed attention: (1) an accurate definition and measurement of MW, (2) the exploration of contextual factors, such as task parameters and participants' arousal and drowsiness levels, and (3) the modality for tracking MW and analyzing pupil correlates. The third point needs special attention in future research, because studying the actual dynamics of MW with physiological measures would add precious information about the physiological processes associated with the onset of MW, its unfolding over time, and its end. This is indeed likely true regardless of the physiological measure that is acquired, thus moving well beyond pupillometry.

What is the essence of MW dynamics is a relatively recent issue in this field (Christoff et al. 2016; Mills et al. 2018; Pelagatti, Binda, and Vannucci 2020), and pupil size may indeed be a good measure to foster new knowledge on the subject, provided that the manifold variables that impact pupil size are duly considered. We believe that, following some feasible caution, we will take better advantage of this special method that is pupillometry, for MW research.

## Author Contributions

**Claudia Pelagatti:** conceptualization (equal), visualization (lead), writing – original draft (equal), writing – review and editing (supporting). **Elvio Blini:** conceptualization (equal), visualization (supporting), writing – original draft (equal), writing – review and editing (equal). **Manila Vannucci:** conceptualization (equal), supervision (lead), visualization (supporting), writing – review and editing (equal).

## Conflicts of Interest

The authors declare no conflicts of interest.

## Related Wires Articles

Pupillometry

Revealing visual working memory operations with pupillometry: Encoding, maintenance, and prioritization

Functional benefits of cognitively driven pupil‐size changes

## Data Availability

Data sharing is not applicable to this article as no new data were created or analyzed in this study.
